# Longitudinal trend of urolithiasis incidence rates among world countries during past decades

**DOI:** 10.1186/s12894-023-01336-0

**Published:** 2023-10-16

**Authors:** Nasrin Borumandnia, Payam Fattahi, Atefeh Talebi, Maryam Taheri, Mohammadamin Sabbagh Alvani, Mohammadreza Mafi Balani, Sadra Ashrafi, Hamid Alavimajd

**Affiliations:** 1 Urology and Nephrology Research Centre, Shahid Beheshti University of Medical Sciences, Tehran, Iran; 2 Faculty of Medicine, Shahid Beheshti University of Medical Sciences, Tehran, Iran; 3 British Heart Foundation Cardiovascular Research Centre, University of Glasgow, Glasgow, Scotland; 4 Student Research Committee, Chronic Kidney Disease Research Centre, Shahid Beheshti University of Medical Sciences, Tehran, Iran; 5 Department of Biostatistics, School of Allied Medical Sciences, Shahid Beheshti University of Medical Sciences, Tehran, Iran

**Keywords:** Urolithiasis, GBD study, Longitudinal analysis, Growth mixture model

## Abstract

**Introduction:**

This study explores the trend of urolithiasis in various countries and categorizes the countries in terms of how their urolithiasis incidence rate has changed over time.

**Methods:**

The incidence rate of urolithiasis in 204 countries from 1990 to 2019, extracted from the Global Burden of Disease study, has been analyzed.

**Results:**

According to the results, all regions had experienced an increasing trend in urolithiasis rate, except for Eastern Europe, Central Europe, and Southeast Asia regions (decreasing rates of -71.4, -56.2, and -9.2 per 100000, respectively). Moreover, the Caribbean region had the highest increasing trend of urolithiasis rates, and Central Asia was in the next rank (increasing rate of 48.3 and 34.3 per 100,000, respectively, *p*-value < .05). Also, African regions revealed significant increasing trends over time (*p*-value < 0.05). The outstanding findings in cluster analysis showed that Afghanistan, Andorra, and Comoros had the most decreasing trend in urolithiasis rates over time (decreasing rate of -128.2 per 100000, *p*-value < .001). Cuba, Cyprus, Czechia, the Democratic People's Republic of Korea, Denmark, and Djibouti were in the next rank in terms of decreasing rate (decreasing rate of -92.3 per 100000, *p*-value < .001). In addition, urolithiasis rates in Congo, Eswatini, Gabon, and Grenada have the most increasing trend (increasing rate of 116.1 per 100000, *p*-value < .001).

**Conclusion:**

The trend of urolithiasis rates was significantly increased in most countries, and Congo, Eswatini, Gabon, and Grenada had the highest trend among others. Also, Afghanistan, Andorra, and Comoros revealed the most decreasing rates, and the trend has dropped remarkably in several other countries.

## Introduction

Urolithiasis, defined as the concentration of minerals in renal calyces and pelvic, is a common and painful urological condition with a significant disease burden worldwide. The prevalence of urolithiasis and, as a result, kidney stone burden has been increasing significantly in recent years all over the world, especially in developed countries [1, 2]. Urolithiasis prevalence in the United States is reported to be increased and reached 8.4%, and men are shown to be susceptible [3]. Urolithiasis causes a significant burden on patients and society; for example, in 2006, United States kidney stone related costs were estimated to be about 10 B USD [4]. Also, urolithiasis is the reason for 4 to 8 percent of end-stage renal disease. Some recent studies show urolithiasis disease distribution changes over the last years [5]. Various factors have been proposed for this phenomenon. Climate and geographical changes, and occupational risks have been known as influential factors [6]. It seems that there is not a similar global trend in urolithiasis rate in the world countries due to several contributing factors. Our literature review revealed an absence of comprehensive studies that cover all countries in terms of exploring the trend of urolithiasis during past decades [7, 8]. This study aims to fill this gap in the literature by providing a comprehensive and updated analysis of the longitudinal trend and rate of urolithiasis among worldwide countries from 1990 to 2019, using the data from the Global Burden of Diseases (GBD). Several studies have utilized the data from the (GBD) study to analyze the global burden of urolithiasis from 1990 to 2019 [9–11]. However, these studies did not classify countries based on changes in urolithiasis incidence rates over time, but rather compared rates among different regions or countries.

To a better understanding of the global epidemiology of urolithiasis and its implications for public health and clinical practice, this study categorizes the countries into different groups based on how their urolithiasis incidence rate has changed over time. This study presents a longitudinal analysis through latent growth modeling on urolithiasis rates in 204 countries and territories. The analytical approach used in the present study is different from previous published studies and is a powerful tool for analyzing longitudinal data, as it can capture the dynamic and complex patterns of change over time and reveal the underlying structure and heterogeneity of the data.

## Methods

Data for incidence rates of urolithiasis (per 100000 persons) in 204 countries and territories were derived from the GBD study [12]. The information used in the present study includes incidence rates of urolithiasis from 1990 to 2019, every two years. We explore the trend of incidence rates of urolithiasis among regions which was designed by the Institute for Health Metrics and Evaluation (IHME), including Andean Latin America, Australasia, Caribbean, Central Asia, Central Europe, Central Latin America, Central Sub-Saharan Africa, East Asia, Eastern Europe, Eastern Sub-Saharan Africa, High-income Asia Pacific, High-income North America, North Africa and Middle East, Oceania, South Asia, Southeast Asia, Southern Latin America, Southern Sub-Saharan Africa, Tropical Latin America, Western Europe, Western Sub-Saharan Africa. In addition, 204 countries and territories were classified into subgroups with similar trends over the years. This study was approved by Shahid Beheshti University of Medical Sciences (Ethic number: IR.SBMU.RETECH.REC.1399.821). All methods were carried out in accordance with relevant guidelines and regulations.

### Statistical analysis

The incidence rates of urolithiasis in each region were described with mean and standard deviation and appropriate plots. The response variable in this study is urolithiasis’s incidence rates, which were modeled using the Latent Growth model to assess the trend in IMHE regions. Also, Growth mixture models (GMM) were applied, and countries were classified into subgroups in which samples within each subgroup followed similar trends over time. GMM is an advanced statistical approach used for trend analysis, and it can take into account heterogeneity in trends among countries. Therefore, using GMM, subgroups of countries are specified, in which countries within each group have similar trends of urolithiasis rates over the period of study. The coefficients of this model, intercept and slope, are interoperated as the overall mean level of the initial outcome and the average rate of outcome change over time, respectively. Statistical analysis was done using M-plus software, version 6.12 (www.statmodel.com).

## Results

**Fig. 1:**
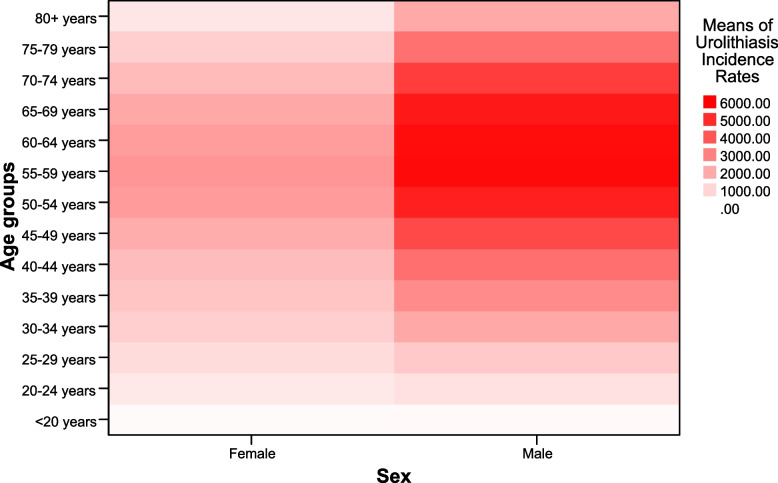
The heatmap shows the means of urolithiasis incidence rate in different age groups and sexes

The heatmap in Fig. 1, shows the average incidence rates of urolithiasis incidence rates during past decades in different age groups and sexes. The heat map uses different shades of red to indicate the incidence rates, with darker red for higher rates and lighter pink for lower rates. The incidence rates are highest in the 6^th^ and 7^th^ decades of life and are lowest in the < 20 years age group for both sexes. As expected, the incidence rates are generally higher for males than for females in all age groups. The incidence rates increase with age for both sexes until the 60–64 years age group, and then decrease slightly for the older age groups.

**Fig. 2:**
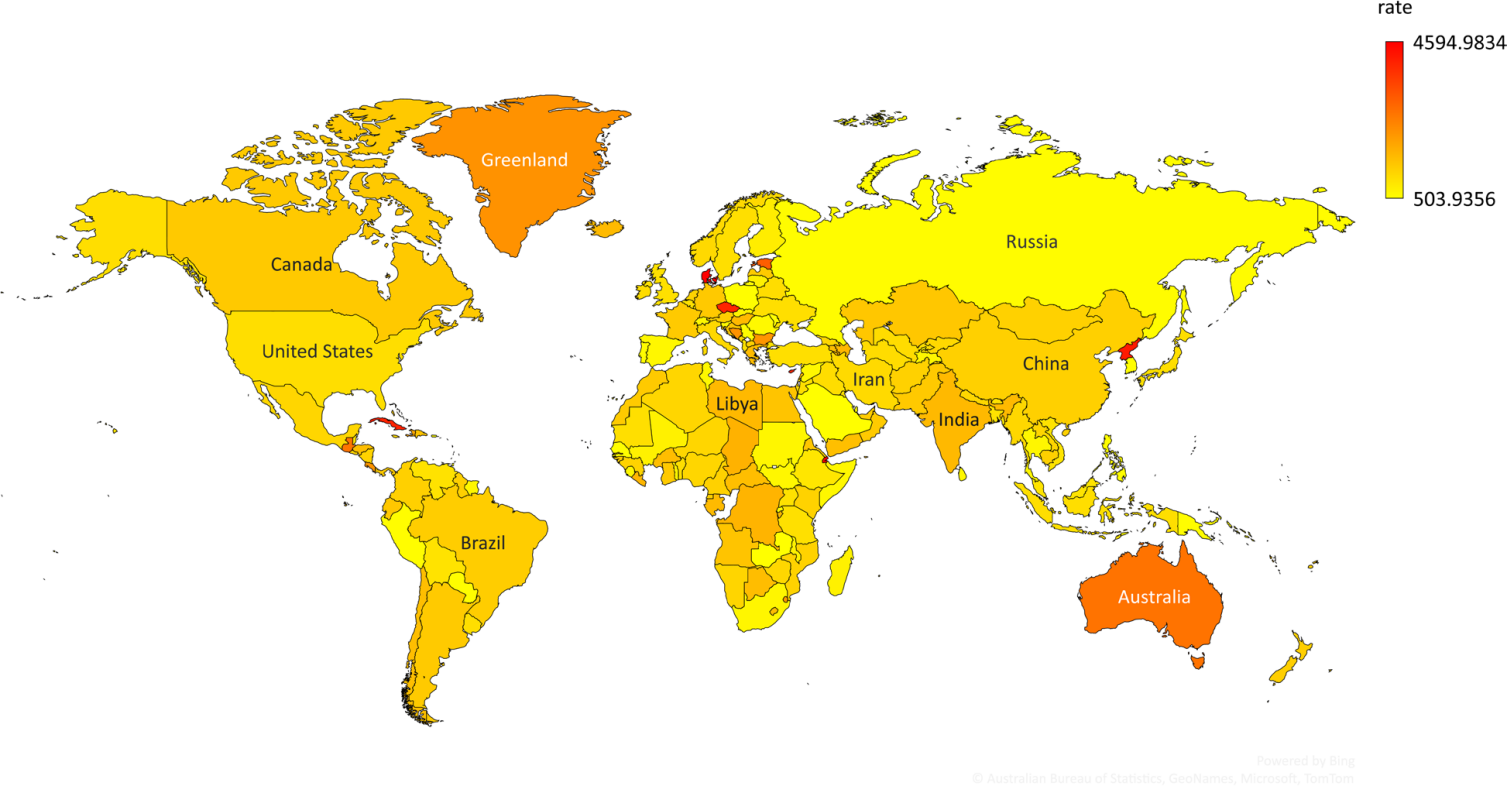
The Geometric means of urolithiasis incidence rates (per 100,000) during past decades in 204 country and regions

The map presented in Fig. 2 shows the distribution of The Geometric means of urolithiasis incidence rates (per 100,000) during the past decades in 204 countries and territories. The descriptive statistics, including mean ± SD of urolithiasis rate, have been presented in Table 1. Regarding this table, the means rates in Eastern Europe countries are considerably distant from the rest of the data. Also, the rates in Eastern Sub-Saharan Africa and Central Sub-Saharan Africa were the lowest among regions.

**Table 1:** The trend of urolithiasis incidence rates (per 100,000) during time

IMHE Regions	1994	1999	2004	2009	2014	2019	Beta^a^	*p*-value
	Mean (SD)	Mean (SD)	Mean (SD)	Mean (SD)	Mean (SD)	Mean (SD)		
Andean Latin America	1522.7 (79.6)	1544.6 (71.8)	1594.2 (24.0)	1693.3 (155.2)	1745.1 (235.2)	1789.9 (202.2)	---^b^	---
Australasia	1425.4 (96.0)	1309.9 (8.0)	1262.5 (18.5)	1253.0 (28.6)	1263.3 (13.7)	1285.3 (4.3)	---	---
Caribbean	1092.5 (116.0)	1133.0 (140.6)	1184.8 (183.3)	1236.0 (231.9)	1275.1 (273.8)	1256.9 (199.2)	48.3	< .001
Central Asia	1702.9 (305.8)	1734.4 (319.1)	1764.4 (359.2)	1763.9 (399.5)	1805.5 (460.5)	1776.1 (361.5)	34.3	0.034
Central Europe	1490.0 (315.8)	1496.4 (330.6)	1642.5 (443.5)	1575.7 (460.4)	1491.3 (371.3)	1281.7 (140.0)	-56.2	.019
Central Latin America	792.0 (93.9)	783.2 (94.0)	777.5 (94.8)	786.3 (89.5)	804.2 (106.1)	833.0 (159.2)	---	---
Central Sub-Saharan Africa	524.9 (5.9)	521.9 (5.7)	535.9 (6.5)	548.8 (7.0)	555.5 (8.2)	577.6 (6.4)	13.7	< .001
East Asia	1256.9 (378.0)	1192.6 (283.4)	1139.1 (178.0)	1035.5 (63.7)	1103.2 (252.2)	1119.4 (295.7)	---	---
Eastern Europe	4184.2 (1283.6)	4029.8 (1202.5)	3703.5 (1049.7)	3555.0 (977.8)	3506.4 (963.0)	3768.0 (1045.6)	-71.4	.132
Eastern Sub-Saharan Africa	517.5 (26.2)	515.2 (23.4)	522.4 (20.5)	533.1 (24.5)	535.5 (24.5)	549.8 (24.2)	8.5	< .001
High-income Asia Pacific	2015.8 (1000.1)	2033.6 (1085.8)	2019.3 (1169.2)	2028.1 (1176.7)	2039.8 (1169.7)	2090.6 (1218.7)	---	---
High-income North America	1134.8 (260.4)	1083.0 (158.8)	1008.7 (23.3)	973.7 (78.3)	977.3 (84.0)	1015.3 (41.6)	---	---
North Africa and Middle East	1151.1 (45.3)	1166.7 (45.4)	1179.0 (46.7)	1211.1 (112.7)	1231.0 (128.8)	1267.9 (131.3)	15.3	< .001
Oceania	993.0 (42.5)	1001.2 (39.8)	995.6 (32.5)	1004.3 (29.1)	1005.7 (23.9)	1043.6 (18.4)	1.8	.264
South Asia	1351.2 (96.4)	1361.0 (78.8)	1384.2 (67.4)	1442.7 (142.5)	1468.8 (147.4)	1559.6 (150.30	---	---
Southeast Asia	1587.1 (457.3)	1579.8 (444.8)	1565.6 (431.4)	1551.4 (423.7)	1566.2 (458.7)	1580.3 (391.5)	-9.2	.695
Southern Latin America	1681.7 (117.5)	1669.3 (91.3)	1628.8 (3.8)	1591.1 (67.3)	1553.9 (144.4)	1679.3 (16.7)	---	---
Southern Sub-Saharan Africa	656.4 (32.2)	647.8 (35.5)	653.5 (34.8)	660.6(31.7)	668.0 (25.9)	684.5 (28.3)	5.9	.001
Tropical Latin America	984.6 (75.5)	994.0 (71.2)	998.0 (70.3)	964.2(20.8)	956.2 (11.6)	965.895.8)	---	---
Western Europe	1532.4 (492.4)	1565.2 (554.4)	1648.2 (659.2)	1659.2 (664.0)	1640.3 (600.7)	1499.0 (327.0)	17.5	.361
Western Sub-Saharan Africa	644.7 (89.6)	637.8 (89.2)	654.6 (89.4)	677.5 (96.5)	679.2 (101.4)	706.1 (72.4)	15.4	< .001
Global	1249.3 (761.4)	1251.7 (746.4)	1266.2 (721.6)	1269.9 (701.2)	1272.9 (688.0)	1269.8 (677.2)	6.5	.222

^a^Trend coefficient which shows the mean change rate per 100,000, in every 5 years, from year 1994 to 2019

^b^Not calculated due to small number of countries in the region

We also applied the LGM to assess the trend of urolithiasis rates in each region separately. According to the obtained Beta from LGM, all regions had experienced an increasing trend of urolithiasis rate, except for Eastern Europe, Central Europe, and Southeast Asia region, which had a negative coefficient. According to the results, countries in Eastern Europe had a mean decrease of 71.4 per 100,000 from the year 1994 to 2019. In the next rank, Central Europe countries reveal a mean reduction of 56.2 per 100,000 during the study period. Moreover, the results show that the Caribbean region had the highest increasing trend of urolithiasis rates (48.3 per 100,000). The countries in Central Asia were in the next rank with a growing trend of 34.3 per 100000 people. African regions, including Western Sub-Saharan Africa (15.4 per 100,000), North Africa and Middle East (15.3 per 100,000), Central Sub-Saharan Africa (13.7 per 100,000), Eastern Sub-Saharan Africa (8.5 per 100,000) and Southern Sub-Saharan Africa (5.9 per 100,000) revealed significant increasing trends over time (*P* < 0.05).

It is notable that, in fact, there are different trends within the countries’ trajectories, and the reported numbers in Table 1 summarize the average. Therefore, we utilized the GMM to classify the countries according to their incidence rate trends over time.

Table 2 provides the estimated result from fitting the GMM to these data. The second column in Table 2 shows the clustering of 204 countries based on their trend of urolithiasis rate. The last column shows the countries which have been included in each cluster. A GMM model with seven linear classes was the best fitting for the data. The linear class means that the trend has been linear at all times. In GMM models, the quality of membership classification was determined using entropy statistics. The entropy statistics were 0.987, which reveals a good quality of clustering.

**Table 2:** Results of growth mixture model for clustering of countries based on their trend of urolithiasis rate along time

Classes	Coefficients of GMM			Countries in each class
	Intercept^a^	Slop^b^	*p*-value	
Class 1: Sharp decreasing trend	2330.26	-128.2	< .001	Afghanistan, Andorra, Comoros
Class 2: Moderate decreasing trend	4808.6	-92.3	< .001	Cuba, Cyprus, Czechia, Democratic People's Republic of Korea, Denmark, Djibouti
Class 3: Slow decreasing trend	1737.8	-42.45	< .001	Azerbaijan, Brazil, Brunei Darussalam, Canada, Chile, Ethiopia, Greece, Iceland, Iran
Class 4: Very slow increasing trend	1011.2	3.5	< .001	Other countries
Class 5: Slow increasing trend	2969.6	13.05	.280	Australia, Democratic Republic of the Congo, Estonia, Greenland
Class 6: Moderate increasing trend	1443.6	56.85	< .001	Bahrain, Bosnia and Herzegovina, Bulgaria, Chad, Cook Islands, Costa Rica, Croatia, Guatemala, Honduras, Kazakhstan, Lebanon, Liberia, Morocco, Yemen
Class 7: Sharp increasing trend	1174.6	116.1	< .001	Congo, Eswatini, Gabon, Grenada

^a^The intercepts represent the estimated overall mean level of the initial urolithiasis rate

^b^The slopes show the average rate of change in urolithiasis rate over time within each class

The intercept coefficients represent the estimated overall mean level of the initial urolithiasis rate in each cluster, and the slopes show the average urolithiasis rate change over time. A positive and negative slope reveals that the rate had an increasing and decreasing trend over time, respectively. For instance, the estimates for the first cluster (intercept = 2330.26, slope = -128.2) reveal that the initial rate of urolithiasis in the countries including this cluster, Afghanistan, Andorra, and Comoros, has been 2330.26 per 100000 in 1990, and it has a decreasing trend with a slope of -128.2 until 2019, every two years (*P*-value < 0.001). Also, countries in cluster 2, including Cuba, Cyprus, Czechia, Democratic People's Republic of Korea, Denmark, and Djibouti, have an initial rate of urolithiasis of 4808.6 per 100000 in 1990, and they have a decreasing trend with a slope of about -92.3 until 2019 (*P*-value < 0.001). Azerbaijan, Brazil, Brunei Darussalam, Canada, Chile, Ethiopia, Greece, Iceland, and Iran entered in class 3, had the next rank in terms of decreasing rate (-42.45 per 100000, *P*-value < 0.001). Countries in clusters 1, 2, and 3 can be defined as having a sharp, moderate, and slow decreasing trend in urolithiasis rates over time, respectively.

**Fig. 3:**
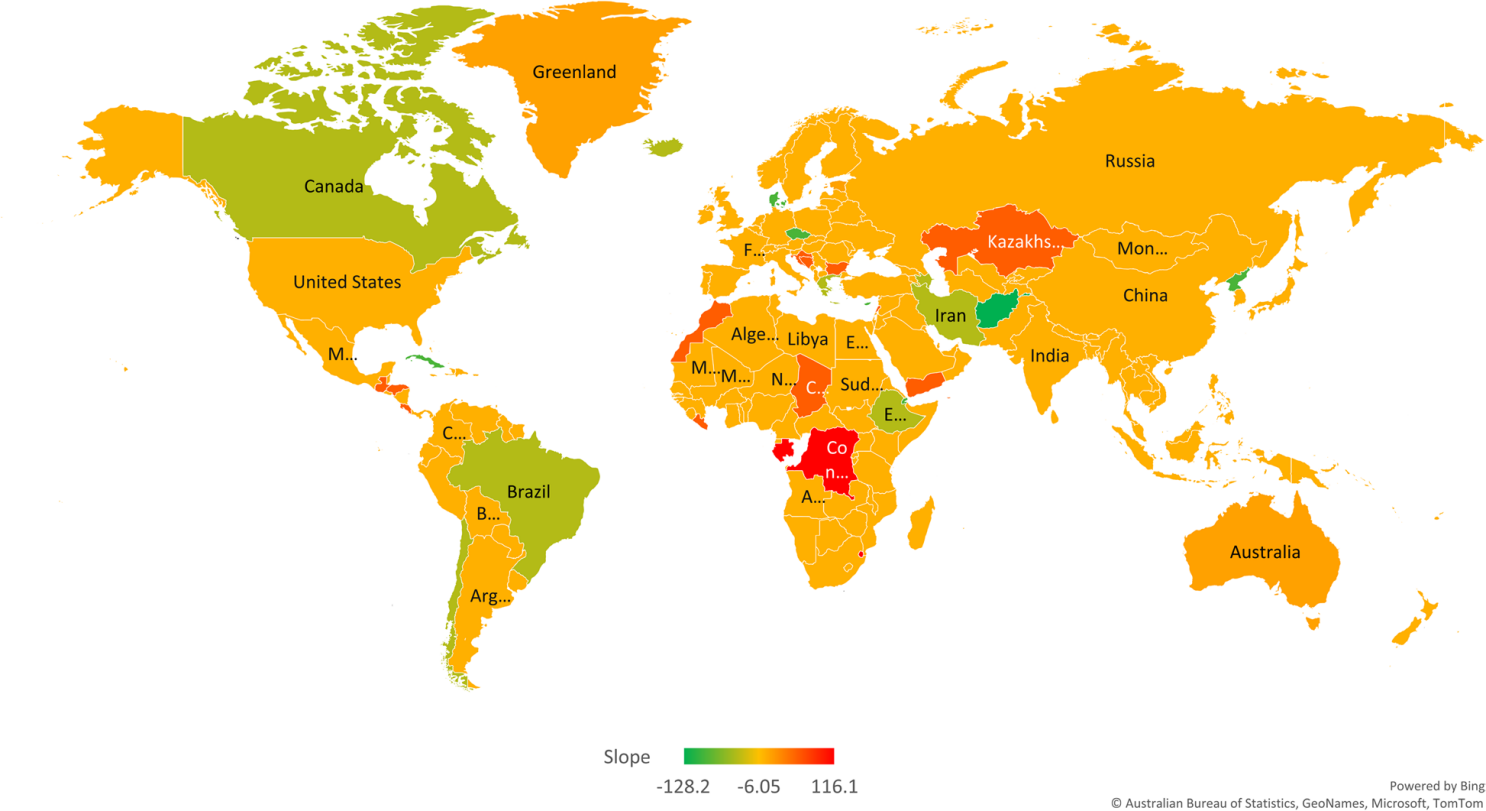
World’s cluster map based on urolithiasis incidence rate trends during 1990–2019. This map shows the result of our own Growth mixture models analysis

In addition, countries in classes 4 and 5 have a slowly increasing trend of urolithiasis over time until 2019. urolithiasis rates in countries included in cluster number 6 (Bahrain, Bosnia and Herzegovina, Bulgaria, Chad, Cook Islands, Costa Rica, Croatia, Guatemala, Honduras, Kazakhstan, Lebanon, Liberia, Morocco, Yemen) have a moderate increasing trend (56.85 per 100000, *P*-value < 0.001). Finally, countries in cluster 7, Congo, Eswatini, Gabon, and Grenada, have experienced a sharp growing trend of urolithiasis rate during this period of time (116.1 per 100,000, *P*-value < 0.001). The colored map in Fig. 3 indicates the estimated trend for the clusters obtained from GMM. Countries with similar color had similar trend of urolithiasis rate.

## Discussion

Some recent studies show changes in the distribution of urolithiasis disease over the few last years [5, 10]. This study explored the incidence of urolithiasis in 204 countries during the last decades. We have investigated the urolithiasis rates among various regions across the world. Also, we clustered countries into subgroups, in which countries within each group had similar trends of urolithiasis rates over the study period 1990–2019.

According to our data, Eastern Europe countries and also countries in central Europe had a considerably decreasing trend of urolithiasis rate during past decades. However, differences in health care systems cause regional differences, but the overall trend is decreasing. This result is in line with Jacob Lang et al., which showed that Eastern Europe had a higher average annual percentage change of urolithiasis than other regions [10]. However, further study claims that the kidney stone has increased markedly in European nations and other industrialized countries during the last decades which contradicts our results [13–15]. We could not find a preventive program whose implementation caused this reduction. But changing social conditions such as changes in lifestyle, eating habits, and physical activity may be important in this context. There seems to be little research on primary urolithiasis prevention [16]. No single preventive program has been found that covers all aspects of urolithiasis prevention. Different types of interventions are needed to address the various causes and complications of urolithiasis. Some of the main interventions that can be suggested to be proposed in current preventive programs are metabolic evaluation and recurrence prevention, infection control and management, genetic counselling and screening, education and awareness, as well as dietary, lifestyle, and environmental factors [17, 18]. Metabolic evaluation and recurrence prevention involves finding out the metabolic imbalances and risk factors that make a person prone to developing stones, and also prescribing suitable drugs or diets to fix them. For example, drinking more water, eating less salt, making the urine less acidic, or taking certain medications can help prevent calcium oxalate or uric acid stones, which are very common [19]. Detecting and treating any urinary tract infections that can lead to or worsen urolithiasis, especially in cases of struvite or infection stones [17]. Genetic counselling and screening through using molecular techniques, biochemical assays, or family history analysis, can help identifying and testing for genetic disorders relating to urolithiasis, especially in cases of cystine or rare stones [19, 20]. Increasing the education and awareness through providing information and guidance to patients and the general public about the causes, symptoms, diagnosis, treatment, and prevention of urolithiasis [17, 21]. Using leaflets, posters, websites, social media, or mass media campaigns are some of the common strategies for raising awareness and promoting prevention of urolithiasis. A comprehensive preventive program, which include all mentioned points could have a more significant impact on the overall burden of urolithiasis, and improve the quality of life and health outcomes of stone formers and reduce the economic costs associated with urolithiasis.

Our results show that the urolithiasis rate in African regions increased over time. There were few studies on the epidemiology of urolithiasis in African countries [22]. We found one study that investigated the trend of kidney study in this area, and our results were consistent with their finding [10].

Based on our results, the global trend of urolithiasis rate was increasing with an increasing rate of 6.5 every five years. The study on urolithiasis average annual percentage change rate, demonstrated a decreasing average annual percentage change rate [10]. The increasing prevalence of nephrolithiasis might be due to the availability of new and more accurate diagnostic tools contributing to an increased diagnosis [23].

Our results showed that countries were classified into 7 clusters based on the trend of urolithiasis incidence. Since urolithiasis is a multifactorial disease, countries in different parts of the world have shown a similar trend of urolithiasis. For example, Afghanistan, Andorra, and Comoros countries (Dedicated to the first cluster in Table 2) have a rapid decline in urolithiasis, even though they are on different continents and even in different climatic conditions. These countries have a high incidence rate of urolithiasis, with a sharp downward trend. These are developing countries which may have similar lifestyles in terms of nutrition and physical activity. However, Afghanistan may be in this cluster due to an incomplete data registry system. Also, based on the results, countries with a moderate and low decline in urolithiasis (countries clustered in class 2 and in the next rank class, 3, presented in Table 2) are located on different continents. However, the common point between them is that some are close to the sea, the weather is mild, and the climate temperate is year-round. Given that ambient temperature is an effective factor for nephrolithiasis, a similar trend of stone kidneys in these countries seems to be correct [24].

Most countries had an increasing trend of Urolithiasis rates (Clusters number 4 to 7). Congo, Eswatini, Gabon, and Grenada have the sharpest increasing rates of urolithiasis. In the next rank, Bahrain, Bosnia and Herzegovina, Bulgaria, Chad, Cook Islands, Costa Rica, Croatia, Guatemala, Honduras, Kazakhstan, Lebanon, Liberia, Morocco, and Yemen had the most increasing rate of urolithiasis. Other countries not mentioned so far have also seen an increase in the Urolithiasis incidence rate. These findings are in line with other studies [7]. These countries are from different continents with various environments and climates. They do not have considerable similarities in social conditions, showing how the multifactor is associated with urolithiasis. Global warming seems to be a common factor among countries. Studies in Arab countries show that their remarkably hot environment and climate are associated with developing nephrolithiasis for most of the year. The studies have showed the role of climate on urolithiasis [25, 26]. It is established that with increasing temperature in an exact area, the prevalence of urolithiasis also increases, and the peak incidence of calculus formation was seen in the hot season [27]. As a result, an epidemic of stone formation can be on the way [28, 29]. Besides many contributing factors, the improvement of socioeconomic conditions unarguably affects this subject. Observations show a very high prevalence of urolithiasis in the wealthier countries of the middle east, like the United Arab Emirates and Saudi Arabia, in contrast to their less affluent neighbors living under the same environmental and cultural conditions [30].

It is notable that gender, race, and median age of the population are important factors that can influence the prevalence of urolithiasis in different countries.

According to a recent review, the prevalence of kidney stones is increasing and historically more common in males [31]. However, recent evidence questions if this gender gap is closing. Changes in diet, obesity rates, metabolic syndrome, and urinary tract infections among women could be factors. Further research and clinical management are needed to understand gender differences in kidney stones.

There is evidence that urolithiasis varies among different racial and ethnic groups [32]. Traditional urinary physicochemical risk factors may not fully explain these differences. Factors such as genetics, environment, diet, and lifestyle may contribute to the racial and ethnic variation of urolithiasis. For instance, a study found that white and Hispanic populations have a higher prevalence of urolithiasis compared to black and Asian populations in the US. The prevalence of urolithiasis has also increased more prominently among women and African Americans in recent years [33].

Urolithiasis is known to increase with age [31, 33]. However, some studies show a rise in urolithiasis among younger age groups, possibly due to obesity, diabetes, and metabolic syndrome in children and adolescents [31]. Therefore, age distribution may influence urolithiasis trends in different countries.

Finally, it is notable that due to the availability of new and more accurate diagnostic tools contributing to an increased diagnosis, asymptomatic stones are more detected due to the more frequent use of high-resolution imaging techniques [23, 34]. On the other hand, some studies have claimed that these data are usually based on hospitalized patients, not those not requiring hospital treatment, making less than 10% of all stone episodes [26].

### Potential biases and limitations

However, the GBD is a comprehensive and systematic effort to estimate the burden of diseases and injuries for 204 countries and territories from 1990 to 2019, it also faces several challenges and uncertainties in the data collection which can introduce potential biases and limitations in the results. One of the main challenges is the variation in healthcare infrastructure, diagnosis practices, and data reporting among different countries. These factors can affect the accuracy and comparability of urolithiasis incidence rates across countries. For example, some countries may have more advanced diagnostic tools or more frequent screening programs than others, which can lead to higher detection rates of urolithiasis. Similarly, some countries may have more reliable and comprehensive data sources or more consistent definitions and classifications of urolithiasis than others, which can influence the quality and comparability of the data. These factors can introduce bias in the data, so caution is advised in interpreting and comparing the results across countries and over time.

## Conclusion

Globally, the incidence rate of urolithiasis has increased during 1990–2019, with various patterns in countries and regions. The trend of urolithiasis rates was significantly increased in most countries, and Congo, Eswatini, Gabon, and Grenada had the highest trend among others. Also, the trend has dropped remarkably in several other countries. Afghanistan, Andorra, and Comoros revealed the most decreasing rates. Overall, while the high economic and health burden of urolithiasis, its rate does not seem to have dropped remarkably in most countries. Therefore, nowadays, when lifestyle leads people to this disease, it is important to implement comprehensive preventive programs that consider controllable risks, including nutritional factors, nutritional deficiencies, lifestyle factors, etc.

## Data Availability

Data is available from http://ghdx.healthdata.org/gbd-results-tool and is freely download.

## Abbreviations

GBD: Global Burden of Disease
IMHE: Institute for Health Metrics and Evaluation
GMM: Growth Mixture Model
